# Proteomics of Brucella

**DOI:** 10.3390/proteomes8020008

**Published:** 2020-04-22

**Authors:** Ansgar Poetsch, María Inés Marchesini

**Affiliations:** 1 Center for Marine and Molecular Biotechnology, QNLM, Qingdao 266237, China; 2 College of Marine Life Sciences, Ocean University of China, Qingdao 266100, China; 3 Plant Biochemistry, Ruhr University Bochum, 44803 Bochum, Germany; 4 Instituto de Investigaciones Biotecnológicas Dr. Rodolfo A. Ugalde, Universidad Nacional de San Martín (IIB-UNSAM-CONICET), San Martín, 1650 Buenos Aires, Argentina

**Keywords:** brucellosis, bacterial virulence, host cell interaction, antibiotic targets, proteogenomics, exoproteome, immunoproteomics

## Abstract

*Brucella* spp. are Gram negative intracellular bacteria responsible for brucellosis, a worldwide distributed zoonosis. A prominent aspect of the Brucella life cycle is its ability to invade, survive and multiply within host cells. Comprehensive approaches, such as proteomics, have aided in unravelling the molecular mechanisms underlying Brucella pathogenesis. Technological and methodological advancements such as increased instrument performance and multiplexed quantification have broadened the range of proteome studies, enabling new and improved analyses, providing deeper and more accurate proteome coverage. Indeed, proteomics has demonstrated its contribution to key research questions in Brucella biology, i.e., immunodominant proteins, host-cell interaction, stress response, antibiotic targets and resistance, protein secretion. Here, we review the proteomics of Brucella with a focus on more recent works and novel findings, ranging from reconfiguration of the intracellular bacterial proteome and studies on proteomic profiles of Brucella infected tissues, to the identification of Brucella extracellular proteins with putative roles in cell signaling and pathogenesis. In conclusion, proteomics has yielded copious new candidates and hypotheses that require future verification. It is expected that proteomics will continue to be an invaluable tool for Brucella and applications will further extend to the currently ill-explored aspects including, among others, protein processing and post-translational modification.

## 1. Introduction

*Brucella* spp. are intracellular Gram-negative pathogens that cause brucellosis, a worldwide distributed infectious disease affecting economically important domestic mammals, wild mammals, and humans. Animals are infected mainly by ingestion of food or water contaminated by infected tissues such as aborted feti or fetal membranes. Human infection generally proceeds via direct contact with blood or tissues from infected animals or by consumption of contaminated dairy products, such as unpasteurized milk and cheese. Brucellosis in animals causes sterility and abortion, resulting in substantial economic losses. In humans, the disease is characterized by high undulating fever in the acute phase, followed by a chronic phase that might affect most organs with manifestations like arthritis, orchitis, hepatitis, encephalomyelitis, and endocarditis. Brucellosis remains endemic in many developing countries in the Middle East, Asia, Africa, and South America, where domestic livestock screening and vaccination programs fail to control and eradicate the disease [1,2,3]. 

The genus *Brucella* currently comprises 12 species and 646 genome assemblies publicly available at NCBI repositories. *B. suis* (pigs), *B. abortus* (cattle) and *B. melitensis* (goats) are the most pathogenic species in humans and have been identified as potential agents of bioterrorism [4]. *Brucella* virulence does not rely on classic virulence factors such as exotoxins, flagella, and capsule. Instead, bacterial pathogenesis largely depends on the ability of *Brucella* to trigger virulence mechanisms and adapt their physiology to the changing environments encountered upon interaction with the host and during the intracellular phases. In this context, one of the key players in *Brucella* virulence is the type IV secretion system (VirB), a macromolecular complex spanning the outer and inner membranes of *Brucella,* required for virulence in a murine model of chronic brucellosis and for intracellular replication in host cell models [5,6,7]. The VirB system is engaged in the delivery of effector proteins into the host cell cytosol, contributing to the control of the intracellular lifestyle of *Brucella* [8,9,10,11,12,13].

Proteomes are defined as the collection of all proteins expressed by a cell type, tissue, organism, etc. Opposite to genomes, proteomes are highly dynamic as proteins are continuously produced, modified, and degraded, depending largely on the physiological conditions and environmental stimuli. Comparative proteomics, combined with genomics and transcriptomics, has proved to be more powerful and has become more popular than classical biochemical methods like In Vivo Expression Technology (IVET) [14] and Signature-Tagged Mutagenesis (STM) [15] for the identification of virulence factors and proteins that contribute to the pathogenesis. In the last two decades, microbiology has benefited from mass spectrometry-based proteomics to characterize the molecular basis of pathogen–host relationships [16,17,18]. 

*B. melitensis* was the first *Brucella* species to be sequenced and the genome became publicly available in 2002 [19]. After completion of the genome sequence, the first proteomic maps were obtained for *B. melitensis* 16M and Rev1 [20,21]. These pioneer proteomic analyses were aimed at providing reference maps for identification of proteins associated to host specificity, virulence, metabolic pathways, and antigenicity. In the following years, proteome studies became more popular and have addressed central topics in brucellosis research. This review will focus on the contribution of proteomics to the identification of novel virulence factors, to study the interaction with the host cell as well as the bacterial responses to environmental stresses. Additionally, we will provide a perspective on the contribution of mass spectrometry-based proteomics to study the subcellular localization of individual proteins and to support the development of better vaccines and diagnostic methods in brucellosis.

## 2. Proteomics Technologies and Their Use for *Brucella*

In the last 20 years, proteomics has been developed at a formidable pace and applied to many areas of *Brucella* research. In the beginning, protein separation by two-dimensional electrophoresis (2-DE), manual spot excision and protein identification by MALDI-TOF (-/TOF) was the method of choice. The earliest works, describing proteomes of different strains and species, though with low coverage, were reviewed in 2002 [19]. With the maturation and improvement of LC-MS-based methods, their use has become more popular and is advantageous for very basic and hydrophobic proteins. Furthermore, LC-MS-based proteomics avoids the technically challenging IEF step of 2D-PAGE and is very versatile regarding sample pre-treatment and fractionation. These advantages translate to a higher number of identified and quantified proteins. Numerous label-using and label-free, MS-based quantification methods have become available. The important features of employed methods and their individual merits have been reviewed extensively in 2012 [22]. Concomitant with analysis techniques, MS data analysis software has matured in the last years [23], now offering robust, label-free quantification and multiplexing of up to 10 samples [24] in one MS run. Since 2012, some of these technological advances have been implemented, yet traditional methods have remained popular in *Brucella* proteomics. There have been frequent uses of 2D-PAGE and MALDI-TOF (-TOF) [25,26,27,28,29,30], sometimes utilizing DIGE to minimize gel-to-gel variation [31,32] or other fluorescent dyes for extended dynamic range [33]. Additionally, the unusual combination of 2D-PAGE with LC-MS/MS has been reported [34]. The multiplexing of samples and the MS/MS-based quantification of iTRAQ reporter ions [35,36], or alternatively with Isotope Coded Protein Labeling (ICPL) [32] has been pursued. In-solution digestion [37,38,39,40,41], or in-gel digestion [42,43], and LC-MS/MS were sometimes employed. 

## 3. Proteomics-based Detection of *Brucella* spp. Immunodominant Proteins 

Immunoproteomics is one of the most common proteomics-based techniques used to identify antigens of potential interest mainly for serodiagnosis and vaccine development. Typically, this technique is based on the fractionation of complex protein samples by 2-DE according to their isoelectric point and molecular mass. After this separation, Western blots with reactive sera are used to detect the immunoreactive proteins, which are then excised from the gel, processed, and identified by mass spectrometry techniques. 

Traditionally, the diagnosis of brucellosis has mainly been based on the detection of anti-smooth LPS (S-LPS) antibodies that induce a very powerful antibody response [44,45,46]. However, an important limitation to this diagnostic method is cross-reactivity with other Gram negative bacteria such as *Yersinia enterocolitica* O:9, *Escherichia coli* O157, *Salmonella typhimurium*, and *Vibrio cholera,* that share high structural similarity with the O polysaccharide epitope of the S-LPS molecule [47,48,49]. Hence, identification of specific immunogenic proteins for the development of LPS-free and protein-based diagnostic methods became a central subject in the brucellosis research of about a decade ago. 

Another application of immunoproteomics is discovery of novel antigen candidates for vaccine design. Brucellosis control and eradication programs largely depend on vaccines, which have been successfully used worldwide for decades. Three vaccines are used in animals: *B. abortus* S19 and RB51, and *B. melitensis* Rev.1 [50,51,52]. They provide effective protection against infection and abortion in animals. However, they exhibit residual human virulence and cannot be used in gravid female animals. Therefore, much effort has been undertaken for the development of safer and more effective subunit vaccines. Several *Brucella* cell surface and intracellular components have been evaluated as protective antigens [53,54,55,56]. Significant activity has been identified for only a few antigens. However, they elicited a poor immunogenic response and protection after *Brucella* infection.

**Figure 1:**
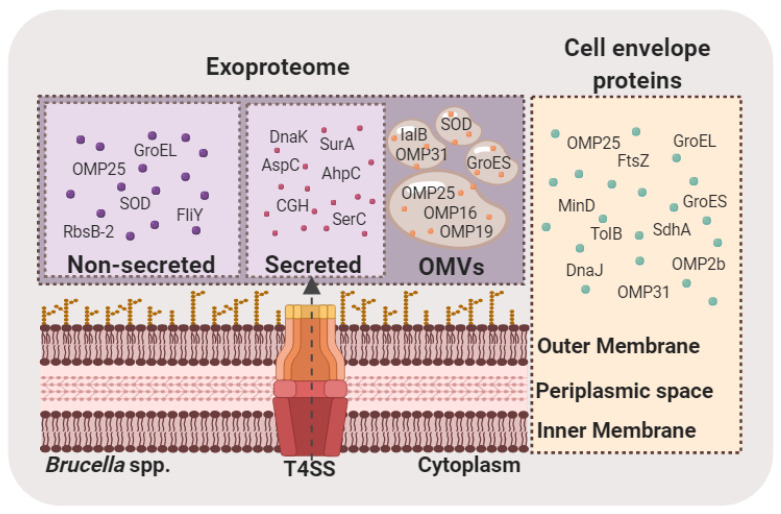
Schematic representation of proteins identified after MS-based analyses in *Brucella* exoproteomes (secreted, non-secreted and inside OMVs) and in the cell envelope (comprising inner membrane, periplasm, and outer membrane). T4SS: type IV secretion system; OMVs: outer membrane vesicles; OMP: outer membrane protein; 2b (BAB1_0660) 25 (BAB1_0722), 16 (BAB1_1707); 19 (BAB1_1930), 31 (BAB1_1639) GroEL: 60 kDa chaperonin (BAB2_0189); SOD: superoxide dismutase (Cu-Zn) (BAB2_0535); FliY: solute-binding protein/glutamate receptor: bacterial extracellular solute-binding protein, family 3 (BAB2_0558); RbsB-2: periplasmic binding protein/LacI transcriptional regulator (BAB2_0377); DnaK: chaperone protein (BAB1_2129); SurA: trigger factor-peptidyl prolyl cis-trans isomerase (BAB1_0917); AspC: aminotransferase (BAB1_1514); CHG: choloylglycine hydrolase (BAB1_1488); SerC: phosphoserine transaminase (BAB1_1699); GroES: 10 kDa chaperonin (BAB2_0190); IalB: invasion protein B (BAB1_0368); FtsZ: cell division protein FtsZ (BAB1_144); MinD: septum site-determining protein (BAB2_0883); TolB: Tol-Pal system protein (BAB1_1709); DnaJ: chaperone protein (BAB1_2130); SdhA: succinate dehydrogenase flavoprotein subunit (BAB1_1901).

Consequently, several proteomic studies were initiated to collect an inventory of *Brucella* immunogenic proteins to be used in serodiagnosis and vaccine development (Table 1). The first studies identified six and eighteen immunodominant proteins in *B. abortus* 1119-3 and *B. abortus* 2308, respectively. Al Dahouk and collaborators aimed at identifying novel *B. abortus* 1119-3 antigens to be used in brucellosis serodiagnosis [45]. The study used 2-DE and Western blots with a rabbit hyperimmune serum for detection of immunodominant spots in *B. abortus* 1119-3 whole cell samples. Immunogenic spots were assigned to proteins: Cu–Zn SOD, BCSP31, ribosomal protein L7/L12, GroEL, GroES, and DnaK. In a similar approach, Connolly and collaborators [57] sought to identify candidate proteins for developing vaccines against *Brucella* infection in humans and cattle. To achieve this, they first identified one hundred and sixty-three proteins in the cell envelope of *B. abortus* 2308 by 2-DE with MALDI-TOF MS and LC-MS/MS. Several new immunogenic proteins such as fumarate reductase flavoprotein subunit, F0F1-type ATP synthase alpha subunit and cysteine synthase A were identified after 2-DE and Western blot analyses probed with antisera from bovine and human infected with *Brucella* (Figure 1). A few years later, the first profiles of *B. melitensis* immunogenic proteins were obtained. Immunoreactive protein spots from whole cell and membrane proteins from *B. melitensis* M5 were identified by Western blotting with bovine, anti-*Brucella*-positive sera and were assigned to 61 proteins by mass spectrometry [58]. Many proteins such as elongation factor G, F0F1 ATP synthase subunit beta, and OMP1were identified for the first time as immunoreactive in *Brucella* and provided novel candidates for vaccine development. Another study by Yang and collaborators described a similar immunoproteomic approach for the development of a brucellosis subunit vaccine [59]. This study identified eleven immunogenic proteins from the total soluble proteome of *B. melitensis* 16M. In 2012, Pajuaba and collaborators used immunoproteomics as a tool to identify antigenic proteins from *B. abortus* S19 that allowed efficient discrimination between infected and vaccinated cattle [60]. In this study, five *B. abortus* S19 proteins (Invasion protein B, Sod, Dps, Ndk, and Bfr) related with antigenicity in naturally infected cattle were identified. In an attempt to analyze time course-dependent immunogenicity against *B. abortus* 544, 2-DE and immunoblotting with sera from mice infected at early, middle and later times post-infection followed by MALDI-TOF MS, allowed identification of seventeen immunodominant proteins [61]. In order to identify new candidate antigens from *B. abortus* RB51, a rough mutant lacking the O-polysaccharide, an immunoproteomics approach by 2-DE and immunoblots with sera from infected cattle was performed [62]. The study identified eleven immunoreactive proteins including Cu/Zn SOD, chaperonin DnaK, chaperonin GroES, cell-division protein FtsZ, 50s ribosomal protein L10 and Invasion protein B that might be helpful in reducing the cross-reactivity in brucellosis serodiagnosis. More recently, Wareth and collaborators analyzed whole cell immunoreactive proteins from *B. melitensis* and *B. abortus* field strains by probing 1D and 2-DE immunoblots with sera from naturally infected ruminant hosts [29,63]. Fifty-two immunodominant proteins were identified that might be useful in the design of alternative serological tests for detection of pan-*Brucella*, *B. abortus*- and *B. melitensis*-specific antibodies.

## 4. Proteomics Strategies in the Study of *Brucella*-Host Cell Interaction and Stress Response

In order to survive and establish a successful infection, intracellular bacteria need to adapt to the harsh conditions of the host cell. Following the entry of the pathogen into the host cell, a reconfiguration of the host and bacterial proteomes takes place, with bacteria producing deleterious virulence factors and the host triggering defense responses. In this context, mass spectrometry-based proteomic techniques have emerged as a valuable tool to monitor and identify bacterial virulence-associated proteins as well as the host proteins involved in the response to the infection. This information has significantly improved the understanding of host–pathogen interactions during the cellular life cycle of pathogens, as recently reviewed [16,17,18,64]. 

Bacterial proteomic analysis upon interaction with the host is challenging as the host proteins need to be efficiently isolated from the limited amounts of bacterial proteins. Some methodologies used to achieve this separation include differential centrifugation, immunomagnetic separation, and fluorescence-activated cell sorting (FACS) [16].

The first study reporting the proteome of *B. suis* recovered from infected murine macrophage-like cells was published by Al Dahouk and collaborators in 2008 [65] (Table 2). To achieve this, a 2-D DIGE analysis was performed at the late stage of *in vitro* infection. After efficient discrimination between host cell and bacterial proteins, 168 proteins, which showed altered concentrations compared to extracellularly grown bacteria, were identified. Most of the proteins were involved in bacterial metabolism and were less abundant in the intracellular condition, suggesting a downregulation of the metabolic pathways participating in energy, nucleic acids, and protein metabolism. In a similar approach, murine RAW264.7 macrophages were infected with virulent *B. abortus* 2308 and the attenuated strain S19, then at different times post-infection, proteomes were compared in order to characterize the physiological adaptation to the intracellular lifestyle of the virulent strain [66]. At early times post-infection, the virulent strain downregulated metabolic and biosynthetic pathways and shifted to low oxygen tension type of respiration. Later, the virulent strain reassumed carbohydrate-based carbon utilization and protein synthesis. The attenuated strain also adjusted its metabolism to face intracellular conditions, but to a lesser extent. Another example of a comparative proteomic approach aimed at identifying *B. abortus* 2308 proteins, which are overexpressed and relevant during the intracellular phase, was performed by Roset and collaborators [67]. Two cyclophilins were identified as overexpressed inside host cells compared to laboratory-grown bacteria. The study also demonstrated that these proteins are required to establish a successful infection. More recently, a quantitative proteomic analysis and identification of *B. abortus* 2308 proteins differentially modulated in vitro and inside host macrophages was performed using iTRAQ isobaric tags [36]. Almost two hundred differentially abundant proteins were identified, undercovering the extensive rearrangement of the bacterial proteome in order to adapt to the intracellular microenvironment within macrophages. 

In order to analyze the changes in *B. suis* 1330 proteome under stress conditions, a comparative study of bacteria grown in anaerobiosis, microaerobiosis or aerobiosis was performed [68]. *Brucella* was shown to resist low oxygen conditions by adjusting the oxidative and non-oxidative metabolisms in order to maintain basic metabolic processes. This adaptation mechanism constitutes an advantage for survival and replication inside the restricted oxygenation environment in the host. Another example of proteomic analysis under stress conditions is illustrated by the study of Cui and collaborators [25]. This work identified the genes regulated by the post-transcriptional regulator Hfq by comparing the proteomes of *B. melitensis* 16M wild type and the *hfq* mutant under stress conditions, known to activate Hfq regulator. As a result, 55 proteins were identified as differentially abundant, demonstrating the role of Hfq in coordination of the adaptive response to stress conditions. Recently, a label-free quantitative proteomic analysis to identify *B. abortus* proteins involved in bacterial response to distinct stresses encountered during its intracellular phase detected more than 1000 differentially expressed proteins belonging to diverse functional groups [38]. 

A few years later, several studies were initiated to address the proteome changes in the host upon infection with *Brucella* spp. A comparative proteomic approach of THP-1-derived macrophages infected or uninfected with *B. abortus* A19 was performed by Wu and collaborators [27]. At different times post-infection, 44 proteins showed differential abundance, highlighting the dynamic proteomic response to the pathogen. Lauer and collaborators used a non-gel-based quantitative iTRAQ mass spectrometry technique to analyze the changes in the protein expression profiles in host cell membrane domains as a response to exposure to rough *B. melitensis* VTRM1 or smooth 16 M [35]. This approach enabled identification of several proteins enriched or depleted in membrane domains engaged in *Brucella* internalization. More recently, a study compared proteomic profiles of bovine trophoblastic cells infected or uninfected with *B. abortus* at early times of infection [69]. This study provided new insights into the interaction between *Brucella* and trophoblastic cells as well as the mechanisms potentially involved in the abortion of feti in infected cattle. Finally, a recent study focused on protein changes in host tissues during infection. Fu and collaborators reported the identification of 12 proteins with significant changes in expression in mice lung tissues after infection via exposure to aerosolized bacteria [70]. 

In order to identify proteins involved in host cell internalization, cell envelope proteins of *B. abortus* mutant strains defective in this process were separated by 2-DE and identified by MS [71]. Deficient internalization in the mutant strains was associated to reduced expression of outer membrane proteins including OMP25, OMP2b and OMP. Recently, a comparative proteomic study was performed to determine the role of c-di-GMP in *Brucella* host cell interactions. To achieve this, proteomic profiles of a phosphodiesterase mutant producing excess c-di-GMP and wild type *B. melitensis* 16M were compared. The analysis revealed that c-di-GMP regulates several key virulence processes, including cell wall and biofilm formation, nutrient acquisition, and the type IV secretion system [37].

## 5. *Brucella* Exoproteome

The term ‘exoproteome’ refers to the protein content found in the extracellular proximity of a given biological system. These proteins are actively secreted to the extracellular medium or are a byproduct of cell lysis and protein degradation. In either way, only the most stable proteins in this environment will remain in abundance. Proteins present in the exoproteome, together with proteins that are displayed on the surface of the bacteria, such as transporters, proteases, toxins, and sensors, are critical for interaction with the environment, including the host cell. 

Secreted proteins usually play, among others, a role in membrane and cell wall biogenesis, pathogenesis, nutrient uptake, and motility. A special type of secreted proteins is contained inside **o**uter **m**embrane **v**esicles (OMVs). OMVs are nanoscale structures secreted by bacteria that carry bacterial surface antigens, small metabolites, nucleic acids, and proteins. These vesicles were shown to participate in the release of virulence factors such as proteases and toxins, signaling, DNA transfer and immunomodulation [72]. 

*Brucella* spp. spontaneously release OMVs that contain LPS, outer membrane proteins and other bacterial components [73,74]. They were shown to promote internalization of *B. abortus* by human monocytes and to downregulate the immune response to favor bacterial persistence within the host [75]. A proteomic approach to investigate the content of *B. melitensis* OMVs identified mostly membrane proteins including outer membrane proteins OMP16, OMP19, OMP25 and OMP31, as well as other immunogenic proteins such as SOD and GroES [76] (Figure 1). Additionally, the study demonstrated the ability of OMVs to stimulate a host cell immune response. 

As already mentioned, VirB secretion system is a major virulence determinant of *Brucella*. Type IV secretion systems (T4SS) are involved in intracellular delivery of effector proteins that modulate host cell functions to promote survival an intracellular replication. Comparative, proteomic-based techniques have been used to identify VirB substrates and proteins affected by the expression and activity of this secretion system. The first study aimed at identifying proteins affected by a highly activated VirB system [77] by comparing proteomes of *B. melitensis* wild type and a *virB* mutant. MALDI-TOF-MS analysis identified differentially abundant proteins involved in diverse functional groups including amino acid transport and metabolism, lipid metabolism, energy production, cell membrane biogenesis, translation, post-translational modifications, and protein turnover. Several virulence-related proteins involved in intracellular survival, including VjbR, DnaK, HtrA, OMP25, and GntR, were down-regulated in the *virB* mutant. Delpino and collaborators sought to identify the role of VirB in the secretion of proteins to the culture medium during vegetative growth [78]. Culture supernatants containing extracellular proteins from *B. abortus* 2308 wild type (WT) and its isogenic *virB10* mutant were harvested and subjected to 2-DE. Differential spots, present in the WT strain but absent in the *virB10* mutant, were considered extracellular proteins secreted in a *virB*-dependent way and were identified by MALDI-TOF analysis. Eleven differential proteins were identified, including DnaK, choloylglycine hydrolase and a peptidyl-prolyl *cis*–*trans* isomerase. To evaluate the effect of VirB system on outer membrane (OM) composition, the OM proteomes from *B. melitensis* wild type and *virB* mutant were isolated and compared [79]. Forty-five differential proteins were identified, most of them comprising outer membrane proteins of the Omp25/Omp31 family. Finally, in order to identify proteins that are produced but cannot be secreted by VirB, *B. abortus* wild type and a *virB* mutant proteomes were compared looking for putative substrates and proteins subjected to T4SS regulation [80]. Sixty-nine differential proteins were identified after 2-DE and MALDI-TOF/TOF MS analysis. The majority were outer membrane and periplasmic proteins, that belonged to diverse functional categories. 

In a recent study, *B. abortus* proteins secreted into the growth medium were identified by 2-DE and MALDI-TOF/TOF analysis. More than twenty-seven proteins including CuZn SOD, OMPs, GroEL and DnaK were identified (Figure 1). Immunization of mice with culture supernatant proteins induced a strong humoral and cell mediated immune responses and exhibited higher protection upon *B. abortus* infection [28]. 

## 6. Antibiotic Targets and Resistance

The lack of effective vaccines against *Brucella* in humans constrains treatment of infections to antibiotics. Unfortunately, the ecological niche of *Brucella* limits the range and efficacy of antibiotics with the consequence of prolonged, often uncomfortable patient treatment with antibiotic combinations to minimize recurrence of infection. Exemplary antibiotic therapies are double treatments with doxycycline+streptomycin/rifampicin, or rifampicin+cotrimoxazole—even triple therapies like doxycycline + rifampicin + cotrimoxazole exist [81].

Importantly, pathogen-specific antibiotics are still sought after, prompting a search for novel, promising targets by employing proteomics. Here, a double strategy has been pursued over the years, i.e., comparative proteomics to unravel antibiotic resistance mechanisms and discovery of key virulence proteins that may serve as potential species-specific antibiotic targets lacking homologs in humans or farm animals.

Rifampicin targets the bacterial DNA-dependent RNA polymerase and inhibits RNA transcription. Rifampicin resistance develops frequently, for the most part manifested as point mutations in the *rpoB* gene encoding RNA polymerase β-subunit [82]. An *in vitro*-generated, Rifampicin-resistant strain with the *rpoB* point mutation V154F was investigated for proteome changes upon exposure to rifampicin [83]. The mutant and original *B. melitensis* biovar abortus strain 2308 were subjected to shotgun proteomics employing N-propionylation (^12^C_3_/^13^C_3_ label) for quantification. Intriguingly, *rpoB* gene mutations affect gene expression on a broader range in various bacteria, and the V154F exchange increased resistance against trimethoprim/sulfamethoxazole, too [83]. For the studied *rpoB* point mutation in *Brucella*, the vast majority of up-regulated (mutant vs. original strain) proteins had functions in basic metabolic pathways (TCA cycle, glycolysis/gluconeogenesis, fatty acid synthesis and metabolism, etc.). A closer look at the cell envelope did not provide any obvious role for transport proteins, such as efflux pumps, in conferring Rifampicin resistance. An increase of an iron ion transporter and catalase suggested that Rifampicin elevated oxidative stress as previously observed in vivo [84]. 

The strategy of subtractive genome/proteome analysis was pursued to identify potential drug (and vaccine) targets to treat *B. abortus* in humans and cattle [85]. The rationale was to single out potentially druggable proteins essential for *B. abortus* that are absent in the host organism. KAAS-KEGG searches [86] retrieved non-homologous proteins in pathogen-specific pathways. For humans as well as cattle, about 40 proteins were identified that were either involved in such pathways or not assigned to any host metabolic pathway. Concerning drug target candidates, the authors intentionally focused on membrane proteins given their proven therapeutic potential. A *Brucella* ABC transporter permease was found as novel drug target in humans not being affected by any known compound in Drugbank, yet its functional importance remained unknown. 

Recently, the *mapB* gene of *Brucella suis* was linked to resistance against both lysozyme and the lipopeptide polymyxin B [40]. By combining several experimental approaches, MapB was assigned functions in cell envelope integrity and as TamB orthologue participating in complex with TamA in protein translocation across the outer membrane. To uncover substrates of MapB and affected other proteins, a Δ*mapB* mutant was compared to the wild type with shotgun proteomics and label-free quantification. The mutant had diminished content of OMP31 family members and OMP25c. Likewise, two inner membrane proteins, an oligopeptide transporter related to β-lactam resistance, and a putative lipoprotein anchoring transpeptidase with putative function in peptidoglycan crosslinking, diminished. From collective proteome, physiological and morphological results, it could be concluded that MapB is not only involved in protein translocation, but is required for proper morphology, cell division and macrophage infection, too.

## 7. Proteogenomics

Typical shotgun proteomics workflows rely for protein identification on sequences determined by genomics or transcriptomics. Then again, proteome data can feed genome annotation/refinement, a procedure commonly named proteogenomics. This has already been done in the earlier proteome studies for *Brucella* and has gained momentum with the considerable improvement in genomics, proteomics, and bioinformatics over the last years.

Already in 2006, for *B. melitensis* and *B. abortus* grown under identical lab conditions, proteomes were compared with the intention to discover proteome signatures explaining the preference for sheep and cattle as respective hosts [87]. By contrasting the Sypro Ruby-stained 2D gels, several importers and secretion systems (Sec, type IV and V) were found as differentially expressed. In light of the high genomic similarity, it appears that differences in expression (regulation) of homologous genes account for adaptation of the two *Brucellae* to their hosts. 

The utility of proteomics to improve genome data was demonstrated for the verification of pseudogenes and correction of translation start sites. NCBI ORF finder [88] detected possible alternative ORFs 200 bp up- or downstream of genes and predicted pseudogenes. In another study, fractionation and shotgun proteomics yielded a comprehensive set of 1729 proteins for the Brucella vaccine strain 104M [42]. Six novel ORFs were identified and three existing ORF models revised. Moreover, identification of N-terminally acetylated peptides allowed to confirm the translation initiation site for 29 genes. The abundance of proteins pertaining to chromosome I was significantly higher than chromosome II.

## 8. Proteases—A Yet Untouched Topic in Proteomics for *Brucella*

Surprisingly, the functional aspects of proteases in *Brucellae* have not been investigated with proteomics so far. This is in sharp contrast to the proven importance of proteases for a great many functions such as host infection and persistence [89,90,91,92], stress response [92,93,94,95,96], morphology [97], communication and signaling [98,99,100], to name the most prominent.

The most studied membrane-residing protease of *Brucella* is Lon. Lon protease contributes to the silencing of macrophages by modulating their cytokine response at an early infection stage [101]. It functions as a stress response protease and its deletion increases sensitivity to puromycin and hydrogen peroxide [93]. The regulatory role was recently probed by comparative RNA-seq of the wild type and *Δlon* strain [92]. Genes involved in stress response, quorum sensing and transcriptional regulation were significantly altered in *Δlon* strain, which substantiates the aforementioned studies. However, proteomics is naturally the method of choice when it comes to the discovery of protease targets and proteolytic processing events. In our previous works with microorganisms, Lon target candidates could be identified by common quantitative shotgun proteomics [102] and more sophisticated proteome turnover analysis [103]. There is no reason to believe why similar studies could not be executed in the future for *Brucella* to illuminate such aspects of protease function.

An ill-characterized protease in bacteria is the rhomboid that cleaves transmembrane helices in the lipid bilayer. In fact, less than a handful of known or potential targets have been described, e.g., halocyanin [104] and tatA. After demonstrating that the *B. abortus* rhomboid is an active protease, we have carried out quantitative shotgun proteomics for *B. abortus* wild type and *Δrhomboid* strain cultivated under defined conditions. A total of 1554 proteins were identified (51.40% of *B. abortus* predicted proteome), from which 82 differed in amount. The integral membrane proteins that evidenced variations were considered as potential rhomboid targets. These included a lytic murein transglycosylase, nitrous-oxide reductase and OmpW (unpublished results). 

## 9. Concluding Remarks

This work is an effort to review the relevant outcomes of proteomic-based studies in *Brucella*. Gel-based and gel-free methods have proved to be irreplaceable tools in the research of multiple aspects of *Brucella* physiology, including virulence. In recent years, throughput and accuracy of mass spectrometric instruments have improved, leading to a significant increase in the amount and quality of available proteomic data. With a large number of proteins involved in *Brucella* host cell interaction and response to stresses being identified, it is now necessary to integrate this information in a way that can be fully exploited by the experts in the field. Nevertheless, there remain proteome features underrepresented in existing *Brucella* studies and worth further exploration, e.g., post-translational modifications and protein interaction.

Future research should focus on the identification of the novel bacteria and host cell proteins involved in crucial steps of the infection, such as adhesion, invasion, replication, and egress from cells. Differences in host specificity and virulence among the different species of *Brucella* also need to be addressed. Studies combining MS-based approaches with other “omics” techniques, like transcriptomics and metabolomics, will certainly allow a better understanding of pathogen-induced changes and immunomodulation of *Brucella*-infected cells and tissues. Moreover, proteomic data combined with genetic studies will provide valuable information on virulence gene expression and post-translational modifications. Proteomics future challenges in the *Brucella* field should be focused on the identification of novel virulence factors and their interconnection with classical virulence determinants, in order to unveil the major traits of *Brucella* pathogenesis, improve the detection methods and identify potential targets for vaccine development, all of which are crucial for the eradication of brucellosis.

**Table 1:** List of proteomics-based studies for identification of immunogenic *Brucella* proteins.

Reference	Species/Strain	Sample	Experimental Design	MS Method	Immunogenic Proteins Identified
[45]	*B. abortus* 1119-3	Whole cell proteins	Identification of immunogenic proteins by 2-D immunoblots probed with rabbit hyperimmune serum against *B. abortus 111-3.*	MALDI-MSnLC-ESI-MS/MS	6
[57]	*B. abortus* 2308	Cell envelope proteins	Detection of immunogenic proteins by 2-D Western blotting with human serum from a *B. suis*-infected patient and with serum from an infected bovine.	MALDI-TOF MSLC-MS/ MS	18
[58]	*B. melitensis* M5	Whole cell and membrane proteins	Identification of immunogenic proteins by 2-D immunoblotting with *Brucella*-infected bovine sera.	MALDI-TOF MS	61
[59]	*B. melitensis* 16M	Whole cell soluble proteins	Identification of antigens recognized by *Brucella*-infected goat and human sera in 2-D immunoblots.	LC–MS/MS	11
[60]	*B. abortus* S19	Antigen preparation obtained after TX-114 extraction	Detection of infection markers by 1-D and 2-D immunoblots probed with sera from naturally infected or S-19-vaccinated cattle.	LC–MS/MS	5
[61]	*B. abortus* 544	Whole cell proteins	Detection of immunodominant proteins by 2-D immunoblots probed with sera from experimentally infected mice.	MALDI-TOF MS	17
[62]	*B. abortus* RB51	Insoluble proteins	Analysis of immunogenic proteins by 2-DE and Western blot with sera from *B. abortus* 2308-infected cattle.	MALDI-TOF/TOF MS	11
[105]	*B. abortus* and *B. melitensis* field strains	Whole cell proteins	Detection of antigenic proteins by SDS–PAGE and Western blotting with sera from naturally infected hosts (cows, buffaloes, sheep, and goats).	MALDI-TOF MS	16
[63]	*B. abortus* and *B. melitensis* field strains	Whole cell proteins	Identification of immunodominant proteins by 2-DE and Western blot with sera from naturally infected hosts (cows, buffaloes, sheep, and goats).	MALDI-TOF MS	36

2-DE: two-dimensional electrophoresis.

**Table 2:** MS-based proteomics studies addressing host-*Brucella* interactions and stress responses.

Reference	Species/Strain	Experimental Design	MS Method	Main Findings
[65]	*B. suis* 1330	2-D-DIGE based analysis of intramacrophagic proteome of *B. suis* at 48 h post-infection, compared to extracellularly grown stationary-phase-bacterial proteome.	MALDI-TOF MS	One hundred and sixty-eight proteins with differential abundance. Most of the proteins identified are involved in metabolic pathways and downregulated intracellularly.
[66]	*B. abortus* 2308 and S19	Comparison of intramacrophagic proteomes of virulent *B. abortus* 2308 and the attenuated strain S19 at different times (0, 3, 20 and 44 h post-infection).	LC MS/MS	At early times post-infection, the virulent strain altered its respiration and downregulated the expression of proteins involved in metabolic and biosynthetic pathways. These changes are reverted to pre-infection levels at 44 h post-infection.
[68]	*B. suis* 1330	2-D-DIGE based comparative analysis of *B. suis* proteome under low oxygen conditions (anaerobiosis and microaerobiosis) and control condition (aerobiosis).	MALDI-TOF MS	Upregulated glycolysis and denitrification in microaerobiosis and anaerobiosis.
[71]	*B. abortus* 1119-3	2-DE-based proteome analysis of cell envelope proteins of mutant strains defective in internalization into host cells.	LC-ESI-MS	Identification of bacterial loci involved in altered expression of cell envelope proteins such as OMP25, OMP2b and OMP28.
[67]	*B. abortus* 2308	2-DE-based comparative proteomic analysis of intracellular and laboratory-grown *B. abortus*.	MALDI-TOF MS	Two cyclophilins were identified as overexpressed during the intracellular phase. The double mutant strain in the genes coding for these proteins is attenuated in cellular and mice infection models.
[25]	*B. melitensis* 16M	2-DE-based comparative proteomic analysis of wild type and *hfq* mutant under stress conditions.	MALDI-TOF/TOF MS	MS identified 55 proteins with differential abundance in the mutant strain. These proteins belong to diverse functional groups including transport and metabolism, outer membrane proteins, post-translational modification and cellular processes.
[27]	*B. abortus* A19	2-DE-based comparative proteomic analysis of THP-1-derived macrophages infected or uninfected with *B. abortus* A19.	MALDI-TOF/TOF MS	MS identified 44 proteins with differential abundance. These proteins were involved in cytoskeleton, signal transduction, energy metabolism, host macromolecular biosynthesis, and stress response.
[35]	*B. melitensis* 16M and VTRM1	Quantitative proteomic approach to study protein redistribution between membrane domains of monocytes exposed or not exposed to *Brucella*.	iTRAQ MALDI TOF/TOF	Several proteins were distinctly enriched or depleted in membrane domains upon exposure to rough and smooth *B. melitensis* strains.
[69]	*B. abortus*2308	2-D DIGE based differential proteomic profile of bovine chorioallantoic membrane explants uninfected and at early stages of infection with *B. abortus*.	MALDI-TOF/TOF MS	Several proteins upregulated during infection are associated with modulation of the innate host immune response to infection with *B. abortus,* including proteins related to TLR signaling and ROS production, as well as proteins associated with inflammation and intracellular trafficking.
[37]	*B. melitensis* 16M	Comparative proteomics approach to identify *Brucella-*specific proteins and pathways affected by changes in bacterial c-di-GMP levels.	LC MS/MS	c-di-GMP levels affect multiple processes related to bacterial virulence, such as nutrient acquisition, cell wall formation, and the type IV secretion system.
[36]	*B. abortus*2308	Comparative proteomic analysis of *B*. *abortus* isolated within the host macrophage cell at late post-infection times and *in vitro*-cultured *Brucella*.	iTRAQ MALDI TOF/TOF	Identification of 197 differentially modulated proteins in intracellular *Brucella*. Many of them were related with iron metabolism, known to play a central role in *Brucella* invasiveness and virulence.
[38]	*B. abortus*104-M	Label-free quantitative proteomic analysis for the identification of proteins involved in stress resistance.	LC MS/MS	Identification of over 1000 differentially abundant proteins under relevant stress conditions. Proteins were included in diverse functional groups such as oxidative phosphorylation, ABC transporters, two-component systems, biosynthesis of secondary metabolites, the citrate cycle, thiamine metabolism, and nitrogen metabolism.
[70]	*B. melitesis* M5	Comparative 2-DE-based proteomic analysis of lung tissue of BALB/c mice uninfected and infected by exposure to aerosolized bacteria.	MALDI TOF/TOF	Identification of 12 proteins differentially expressed in lung tissue during infection. The proteins with increased expression were related to protein transport, antioxidant function, and antiviral or cell activation. Proteins with decreased expression were related to cytoskeletal structure, enzyme activation, or cell intoxication and transformation.

2-DE: two-dimensional electrophoresis.
